# Wrist function recovery course in patients with scaphoid nonunion treated with combined volar bone grafting and a dorsal antegrade headless screw

**DOI:** 10.1186/s13018-020-02055-0

**Published:** 2020-11-10

**Authors:** Chen-Wei Yeh, Cheng-En Hsu, Wei-Chih Wang, Yung-Cheng Chiu

**Affiliations:** 1 School of Medicine, China Medical University, Taichung, 40447 Taiwan; 2 Department of Education, China Medical University Hospital, Taichung, 40447 Taiwan; 3 Sports Recreation and Health Management Continuing Studies-Bachelor’s Degree Completion Program, Tunghai University, Taichung, 407 Taiwan; 4 Department of Orthopedics, Taichung Veterans General Hospital, Taichung, Taiwan; 5 Department of Orthopedic Surgery, China Medical University Hospital, China Medical University, No. 2, Xueshi Rd., North Dist, Taichung City, 40454 Taiwan

**Keywords:** Function recovery, Scaphoid nonunion, Volar approach, Dorsal approach, Bone grafting, Headless screw

## Abstract

**Background:**

Surgical treatment is necessary for scaphoid nonunion. Open surgery with a combined volar and dorsal approach is thought to have poor functional outcomes and a prolonged recovery course. However, the detailed recovery course for this approach is rarely reported. The aim of this study was to investigate the recovery course and radiographic outcome for patients with scaphoid nonunion who underwent a combined volar bone grafting and dorsal antegrade headless screw approach.

**Material and methods:**

Eighteen patients with scaphoid nonunion who underwent combined volar bone grafting and dorsal antegrade headless screw fixation were enrolled in this retrospective study. Preoperative and serial postoperative wrist functional and radiographic outcomes were collected and analysed.

**Results:**

All 18 patients achieved bone union at a mean time of 14.3 weeks. Compared to the preoperative status, the grip strength, wrist motion arc, and Mayo Wrist score were improved significantly 6 months after surgery, whilst the Disabilities of the Arm, Shoulder, and Hand (DASH) score did not recover until 12 months after surgery. Significant improvements were found in all scaphoid radiographic parameters.

**Conclusion:**

The surgical outcomes for scaphoid nonunion treated with a combined volar bone grafting and dorsal antegrade headless screw achieved a high union rate, with great wrist functional and radiographic outcomes. The earliest recovered wrist functional parameters were grip strength, motion arc, Mayo Wrist score and finally the DASH score at postoperative 6 months and 12 months, respectively.

## Introduction

Scaphoid fracture accounts for 60% of all carpal fractures, and be the second most fractures around the wrists [1]. The highest nonunion rate was 15.5% of scaphoid fracture amongst whole body bones [2]. Untreated scaphoid nonunion may progress to scaphoid nonunion advanced collapse, dorsal intercalated segment instability (DISI) deformity, and generalised wrist arthritis. Surgical procedures including bone graft and screw fixation are the gold standard treatments for scaphoid nonunion [3, 4]. In comparison to percutaneous screw fixation and arthroscopic bone grafting, a combined volar and dorsal approach for bone grafting and screw fixation is thought to have inferior functional outcomes and prolonged recovery course because of the risks of a disrupted blood supply and scar formation [5]. However, the detailed recovery course and the functional and radiographic outcomes of this approach are rarely reported in the literature. A better understanding of the recovery course of combined volar and dorsal approaches may fill the gap between clinical science and clinical practice [6–8]. The aim of this study was to investigate the recovery course and the functional and radiographic outcomes of patients with scaphoid nonunion who were treated with combined volar bone grafting and dorsal antegrade headless screw fixation.

## Material and methods

### Study population

The trial was approved by the Research Ethics Committee of the China Medical University Hospital, Taichung, Taiwan (Protocol ID: CMUH109-REC1-093), and was conducted in accordance with the ethical principles of the Helsinki Declaration. Clinical data of 18 patients with scaphoid nonunion who underwent volar bone grafting that included a dorsal antegrade headless screw from January 2016 to June 2019 were collected. The inclusion criteria were scaphoid waist fracture with no sign of bone union for more than a 3-month period, and Herbert classification type D1 (nonunion > 6 weeks, fibrous union with no deformity), D2 (nonunion > 6 weeks, pseudarthrosis with early deformity), D3 (non-union > 6 weeks, sclerotic pseudarthrosis with advanced deformity) [9]. The exclusion criteria included radio-scaphoid arthritis, scaphoid nonunion with AVN, and previous scaphoid surgery.

### Surgical technique

The procedure was performed under general anaesthesia. The patient was positioned supine with the upper limb placed on a radiolucent table. A tourniquet was placed at the upper arm and inflated to 250 mmHg during surgery. A 4 cm curved incision was made along the radial border of flexor carpi radialis tendon (FCR) proximally from the wrist crest and distally to the scaphoid tubercle. The radial artery and its dorsal branch were carefully protected. The capsulotomy was performed above the radial scaphoid joint with a vertical incision. The nonunion site was then exposed. Interposed fibrous tissue and sclerotic bone that had occupied the nonunion site were removed thoroughly. The tourniquet was released to ensure the bleeding viability of the fracture fragment. Two 1.6-mm Kirschner wires were inserted perpendicularly into the central portion of proximal and distal fragments of the fractured scaphoid. The nonunion gap was opened with the aid of a pin distractor. Mercerized cancellous bone harvested from the iliac crest was impacted into the wedge-shape fracture gap. The scaphoid length, humpback deformity of scaphoid, and the DISI deformity of carpal bone were corrected with this method and verified with intraoperative fluoroscopy. The dorsal approach was then adapted for screw fixation. A 3-cm longitudinal incision was made over the ulnar border of the Lister’s tubercle. The extensor retinaculum was incised along extensor pollicis longus tendon (EPL), and the dorsal radiocarpal joint capsule was exposed between the third and fourth extensor compartment. A vertical capsulotomy was made to expose the scapholunate ligament. Care was taken not to disrupt the blood vessels entering the mid-portion of the scaphoid as well as protection the integrity of scapholunate ligament. The wrist joint was positioned to 30° of flexion and 10° of ulnar deviation to expose the screw entry point. A 1.0-mm antegrade Kirschner wire that served as a guidewire was inserted along the central axis of the scaphoid under intraoperative fluoroscopy. With an appropriate screw length, a 3.0 Dartfire screw (Wright, Memphis, TN, USA) was inserted into the scaphoid along the central axis of the guidewire. The fracture and bone graft stability were verified with fluoroscopy after the headless screw fixation. The wound was then closed in layers with gauze packed well and protection with short arm thumb spica cast immediately after surgery.

### Postoperative protocol

A short arm thumb spica cast was applied for 6 weeks and was replaced with a wrist brace for an additional 6 weeks. Clinical and radiographic follow-up were arranged every 4 weeks for the first 3 months. After the short arm thumb spica cast was removed, the patients began to participate in a rehabilitation programme, in which a well-trained physical therapist applied passive motion training. At 12 weeks after surgery, low-impact exercises with muscle strengthening were allowed. The patients were allowed to return to full sports activity 6 months after surgery. Wrist flexion-extension arcs, grip strength, the Visual Analogue Scale (VAS), Mayo Wrist score, and DASH score were recorded at postoperative 3, 6, 9 and 12 months, respectively.

### Radiographic examination

Our protocol was based on the routine scaphoid series recommended by the American College of Radiology in Shenoy et al. [10]. The scaphoid series was taken in four views: posterior-anterior, lateral, semi-pronated oblique, and posterior-anterior ulna deviation.

The radiographic examination was performed at postoperative 1, 3, 6 and 12 months. The definition of bone union was that the bony trabeculae grew over the junction between the bone graft and the distal and proximal fragments according to the four views of the scaphoid series, and the clinical symptoms of pain and tenderness relieved [11, 12]. Bone union on was routinely performed at 6 months postoperatively to confirm the union status, which was defined as bone bridging over 50% on the nonunion site [13, 14]. Radiographic outcomes were evaluated with the following five parameters: scaphoid axial length [15], scapholunate angle (normal range, 30 to 60°; humpback deformity > 60°) [16], radiolunate angle (normal range, 0 to 10°; DISI deformity > 15°) [17, 18], carpal height ratio (normal range, 0.5; carpal collapse < 0.45) [19], and the lateral intrascaphoid angle (normal range, < 35°; humpback deformity > 35°) [20].

The measurements of these five parameters were performed by two experienced hand surgeons. If there was a discrepancy in the measurement value or bone union time, a revised measurement was determined after re-measurement and discussion by the two surgeons.

### Clinical evaluations

Grip strength and flexion-extension arcs were measured by a blinded observer, who was not aware of the surgical plan, and other radiographic findings were measured preoperatively and at postoperative 3, 6, 9 and 12 months, respectively. The hand grip strength was measured with a Jamar Hydraulic Hand Dynamometer (Jamar Technologies/America) using the Southampton protocol as follows [12]: patients were seated with back support and the hips flexed as close to 90° as could be tolerated. The patients rested their forearms on the arms of the bed with their wrists in a neutral position. The measurer supported the weight of the device by resting it on his or her palm. Measurements were performed three times for each hand to give six readings in total. The best of the six grip strength measurements was used in the statistical analyses. The operated hand was measured as a percentage of the normal side. Considering whether the dominant or non-dominant hand was injured, we employed the 10% rule for data correction [13–15]. The active wrist flexion-extension arcs for the operated and non-operated hands were measured with the manual universal goniometer.

The functional outcomes of the 18 patients were evaluated with Visual Analogue Scale (VAS), DASH score and Mayo Wrist score questionnaires preoperatively and at 3, 6, 9 and 12 months postoperatively. The patient’s satisfaction was classified into four degrees according to the Mayo Wrist score: excellent, 90 to 100; good, 80 to 90; satisfactory, 60 to 80; and poor, below 60.

### Statistical analysis

Data analysis was performed using the SPSS software (Version 20.0; Chicago, Illinois). Univariate analysis was performed using frequencies for descriptive statistics. Kruskal-Wallis test was used in the analysis of the categorical variable. Post hoc analysis, and Wilcoxon rank-sum test performed to evaluate the significant differences between preoperative and postoperative measures at 3, 6, 9 and 12 months. Correlations were considered significant if *p* values were less than 0.05 (two-sided).

## Results

From January 2016 to June 2019, 25 patients received surgical treatment for scaphoid nonunion in our hospital. Three were excluded because of avascular necrosis, three due to loss of follow-up, and one for previous scaphoid surgery. Eighteen patients were finally included for analysis (Table 1). All patients received volar bone grafting and a dorsal antegrade headless screw. The patients’ average age was 32.7 (range, 20 to 59) years. Initial injury to operation time was 20.8 (range, 3 to 144) months. Mean time to union was 14.3 (range, 8.9 to 20.9) weeks. Thirteen (72%) patients were men and 5 (28%) were women. Four patients were smokers (22%). The injury mechanism included ten traffic accidents (56%), and eight falls (44%). Scaphoid nonunion on the right side occurred for 9 (50%) and on the left side for 9 (50%), and the dominant hand of all patients was the right side (100%). The fracture site for all patients was at the scaphoid mid-portion. According to the Herbert classification, two cases were D1 (22%), five cases were D2 (28%), and nine were D3 (50%).

**Table 1:** Characteristics of the patients

	Number (%)
Sex	
M	13 (72%)
F	5 (28%)
Side	
R	9 (50%)
L	9 (50%)
Smoking history	
Y	4 (22%)
N	14 (78%)
Dominant	
R	18 (100%)
L	0
Herbert	
D1	2 (11%)
D2	7 (39%)
D3	9 (50%)
D4	0
Injury mechanism	
Traffic accident	10 (56%)
Falling down	8 (44%)

**Fig. 1:**
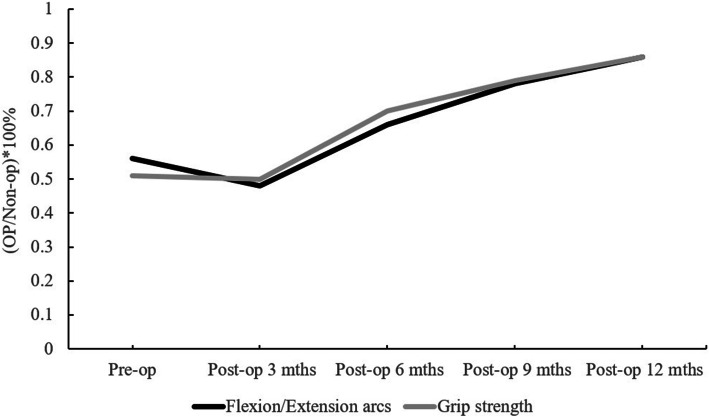
Functional recovery course

The recovery course for the grip strength, arc of motion, DASH score and Mayo Wrist score from the preoperative period to 12 months postoperative is shown in Table 2. The wrist function recovery course after the operation was divided into three phases (Fig. 1). A downswing phase was noted from the preoperative period to 3 months postoperatively. The lowest wrist function status was found at postoperative 3 months. The upswing phase started from 3 to 6 months postoperatively. A prominent wrist function improvement occurred in which low-impact exercises and light activity were allowed under the assistance of an experienced therapist. The improvement of wrist function was reduced during the steady growth phase (6 to 12 months postoperatively).

**Table 2:** Recovery course of functional scores in pre-operative and different post-operative, and radiographic parameters of scaphoid deformity

	Preoperative	Postoperative 3 months	Postoperative 6 months	Postoperative 9 months	Postoperative 12 months	*P* value*
**Functional score**						
Grip strength^a^	51% ± 20%	50% ± 18%	70% ± 16%*	79% ± 10%*	86% ± 12%*	< 0.05
Motion arc^b^	56% ± 10%	48% ± 10%	66% ± 12%	78% ± 13%*	86% ± 11%*	< 0.05
DASH score	32.8 ± 18.1	43.8 ± 21.2	30.9 ± 15.3	19.0 ± 14.2	12.4 ± 11.4*	< 0.05
Mayo Wrist score	42.5 ± 14.6	40.2 ± 15.5	63.6 ± 12.5*	72.8 ± 12.7*	84.4 ± 10.8*	< 0.05
**Radiographic parameters**						
Scaphoid axial length	24.8 ± 6.1		27.2 ± 5.3			< 0.05
Intrascaphoid angle	43.7° ± 6.7°		30° ± 6.4°			< 0.05
Scapholunate angle	61.6° ± 10.6°		51.3° ± 9.9°			< 0.05
Radiolunate angle	25.8° ± 5.5°		11.3° ± 3°			< 0.05
Carpal height ratio	46% ± 4%		52% ± 2%			< 0.05

Grip strength^a^ (op/non-op) × 100%; motion arc^b^ (op/non-op) × 100%

*P value, significance difference under Wilcoxon rank-sum test

**Fig. 2:**
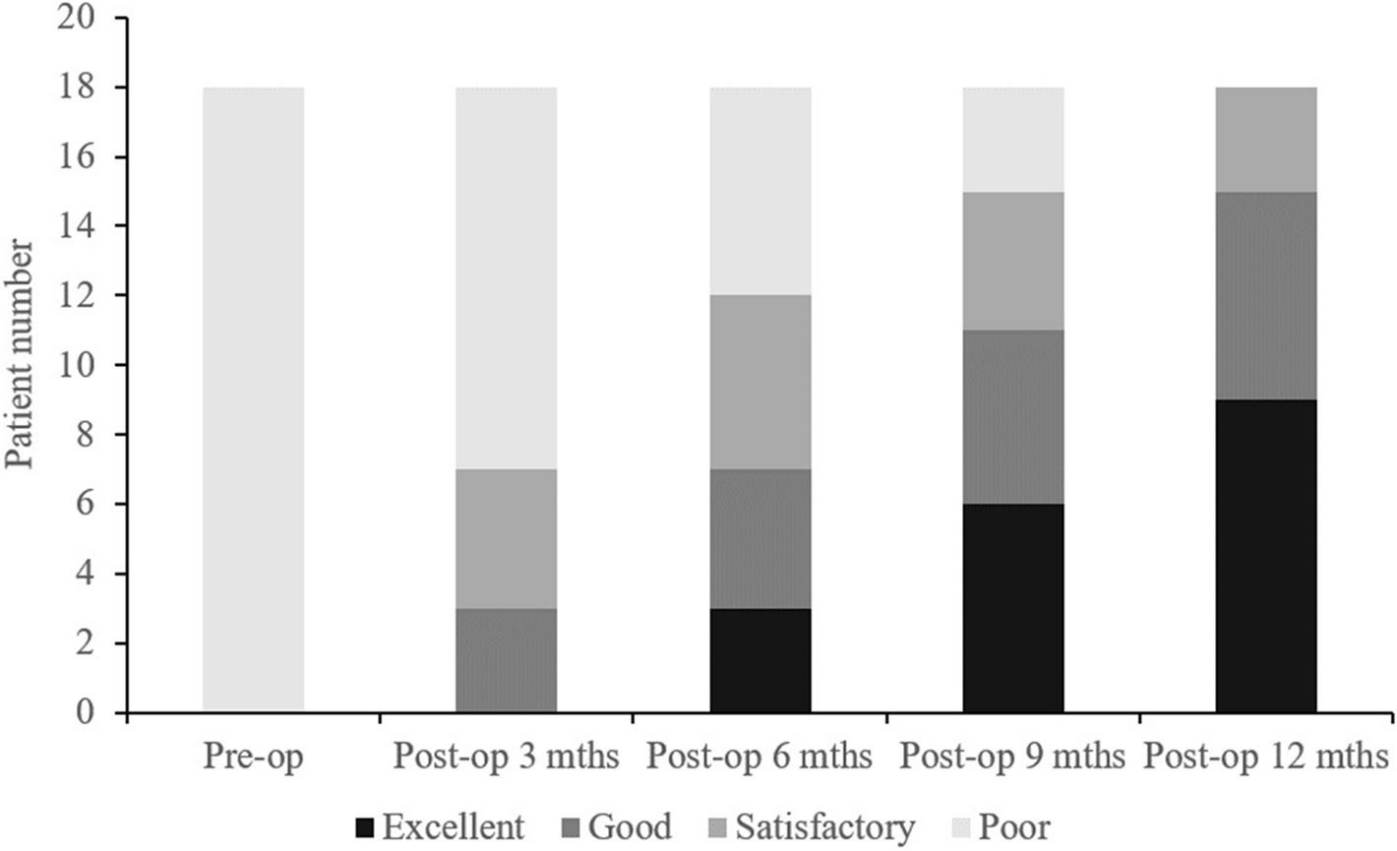
Mayo Wrist score

At postoperative 6 months, the grip strength, Mayo Wrist score (Fig. 2) and motion arcs began to improve significantly compared to preoperative status (Table 2). Finally, the DASH score was significantly improved at postoperative 12 months (*P* < 0.05) (Table 2).

The preoperative and postoperative radiographic parameters are shown in Table 2. Significant postoperative improvements (*P* < 0.001) were found in the radiolunate angle (25.8 to 11.3°), scaphoid axial length (24.8 to 27.2 mm), scapholunate angle (61.6 to 51.3°), lateral intrascaphoid angle (43.7 to 30°), and carpal height ratio: (0.46 to 0.52) compared to the preoperative data.

## Discussion

The purpose of this study was to investigate the recovery course, and the functional and radiographic outcomes of patients with scaphoid nonunion who were treated with combined volar bone grafting and dorsal antegrade headless screw fixation. The main findings of our study are as follows: (1) The surgical outcomes for scaphoid non-union treated with combined volar bone grafting and a dorsal antegrade headless screw achieved a high union rate, satisfactory wrist functional and radiographic outcomes. (2) The wrist functional recovery course after the double approach surgery for scaphoid nonunion was divided in three phases: a downswing phase from operation to postoperative 3 months, an upswing phase from postoperative 3 to 6 months, and a slower progressing phase from 6 to 12 months postoperatively. (3) Compared to the preoperative status, the grip strength, Mayo Wrist score and motion arcs were the earliest recovered wrist function parameters that had significant improvements at 6 months postoperatively, and finally the DASH score at 12 months postoperatively.

Arthroscopic surgery has the advantages of direct visualisation, facilitated debridement of the scaphoid nonunion site, and minimal violation of the scaphoid vascularity [17]. High union rates of approximately 84 to 100% are reported with this method [5, 21–23]. Though this minimally invasive technique has yielded good results with minimal morbidity, its use is still limited to scaphoid nonunion without a large bone defect [24]. In addition to large bone defect filling, the open volar approach is also advantageous in correction to the humpback deformity, scaphoid length and DISI [12, 25] (Table 3).

**Table 3:** Comparison of clinical outcomes and functional score of scaphoid nonunion treated with arthroscopic surgery and open surgery

	No.	Union time (weeks)	Union rates	Cast after surgery (weeks)	Follow-up time (months)	Motion arcs to non-operative side	Strength to non-operative side	Mayo Wrist score (excellent and good)	DASH score
**Arthroscope**									
Kim JP [26]	28	11	86%	4-6	24	Unchanged	89%	44 to 13	23
Kang [27]	33	ND^a^	97%	6	24	100 to 109° (op^c^)	35 to 50 kg (op ^c^)	56 to 89	4
Delgado [28]	13	7	93%	2	17.3	153.6 to 166.1° (op^c^)	ND^a^	ND^a^	8
Lee [29]	27	10	96.3%	10	18	90.1%	89.6%	18/27 (67%)	ND^a^
Liu [30]	25	12	100%	6.6	21	94.2%	ND^a^	95.2 (post-operative)	ND^a^
Oh-A* [25]	28	ND^a^	96.4%	2	24	99.8 to 108° (op^c^)	81.4%	25/28 (89%)	5.6
**Open**									
Oh-O^#^ [25]	34	ND^a^	97.1%	2	24	97.5 to 103.2° (op^c^)	86.1%	28/34 (82%)	6.8
Mani [31]	45	13.15	93%	6	12	80%	101%	40/45 (89%)	ND^a^
Dustmann [32]	52	ND^a^	84.6%	12	8.6	91%	93%	91.2 (post-operative)	9.2
Han [33]	30	12.5	89%	6	37.5	86%	88%	28/30 (93%)	ND^1^
Kim JK [34]	35	12.9	97%	6-8	12	NSD^b^	87%	ND^a^	9.5
Kim J [35]	24	12	92%	6	24	56%	32 to 38 kg (op^c^)	52 to 70	ND^a^

A* arthroscopy cancellous bone graft; *O*^#^ open volar cancellous bone graft; *ND*^a^ no data; *NSD*^b^ no significant difference; *op*^c^ operative

A literature review regarding the wrist functional recovery course after scaphoid nonunion surgery is presented in Table 4 [25–35]. The union rates ranged from 84.6 to 97.1% for open surgeries and 86 to 100% for arthroscopic surgeries. Our patients achieved a 100% union rate, which was higher than that for the open surgery group in previous studies. The high union rate in our study suggested the importance of deformity correction and fixation stability, which were determined by the bone graft quality and the screw position. In addition to the compacted-wedge shape bone graft, the centrally placed screw is the keystone of this procedure. Biomechanically, centrally placed screws have superior stiffness, support a greater load at failure, have a longer screw length and shorter healing time than eccentrically placed screws [36–38]. A centrally placed screw from the volar side can also be achieved by a lever trapezium approach or by drilling a portion of it; however, many surgeons prefer the dorsal approach because of the ease of access and the ability to place a screw closer to the central axis [39, 40]. In addition, a study that has examined scaphoid intraosseous vascular anatomy also shows that the central axis and antegrade dorsal screw fixation cause less disruption of the scaphoid internal blood supply than that for the retrograde volar screw [41]. In our study, the headless screws were inserted through the dorsal mini-open approach instead of a purely dorsal percutaneous technique. A mini-open dorsal approach has been shown to be safer than the purely percutaneous method when approaching from the dorsal mid-portion. Weinberg et al. have shown that there is a 13% chance of tendon injury with a purely percutaneous technique [42]. We believe that the mini-open wound to the dorsal mid-portion provides adequate exposure for vessels, ligament protection, and provides an excellent screw insertion site to allow easy insertion of the central axis screw.

**Table 4:** Comparison of carpal alignment correction in arthroscopic and open bone grafting in the literature

	SAL^a^ (mm)		LISA^b^ (°)		SLA^c^ (°)		RLA^d^ (°)		CHR^e^	
	Pre^f^	Post^g^	Pre^f^	Post^g^	Pre^f^	Post^g^	Pre^f^	Post^g^	Pre^f^	Post^g^
**Arthroscopic bone graft**										
Kim JP [26]	26.5	27.3	42.8	33.5	59.3	51.6	NSD^h^			
Delgado [28]					67.7	47	30.8	4		
Oh-A* [25]			33	26.5	53.3	45.9	7.5	5.2	0.64	0.59
**Open bone graft**										
Oh-O^#^ [25]			39.2	22.6	58.9	46.8	8.8	4.8	0.65	0.55
Mani [31]					49.6	36.2			0.66	0.6
Dustmann [32]					65	55				
Han [33]	22	26	40	32	61	56				
Kim JK [34]			55	35			11.5	5.26	0.72	0.65
Kim J [35]					62	56			0.6	0.57

A* arthroscopy cancellous bone graft; *O*^#^ open volar cancellous bone graft; *SAL*^a^ scaphoid axial length; *LISA*^b^ lateral intrascaphoid angle; *SLA*^c^, scapholunate angle; *RLA*^d^ radiolunate angle; *CHR*^e^, carpal height ratio; *Pre*^f^ preoperative degree; *Post*^g^ postoperative degree; *NSD*^h^ no significant difference

Table 4 shows the final postoperative motion arcs, grip strength and function scores of previous studies. The follow-up time ranged from 8.6 to 37.5 months postoperatively. Most studies had good to excellent results at the final follow-up. However, the detailed recovery course in the first postoperative year was not reported. In our study, we found that the earliest recovered parameters were the grip strength, the Mayo Wrist score and motion arcs, which had recovered significantly at 6 months postoperatively, and finally the DASH score at 12 months. In our protocol, wrist immobilisation with a short arm thumb spica cast and wrist brace was applied for the first three postoperative months. After three postoperative months, low-impact exercises and light activity were allowed under the assistance of an experienced therapist. An average bone union time of 14.3 weeks was observed in this period. In our observation of the functional recovery course of the wrist, the grip strength recovered quickly after the bone union, which could be the early objective predictor to confirm the bone union. The motion arcs improved quickly after the removal of the wrist brace, which could be a reliable parameter to evaluate the intensity and frequency of rehabilitation. The Mayo Wrist score is a questionnaire that consists of pain, satisfaction, range of motion and grip strength. The objective grip strength and motion arcs improvement that occurred in the early postoperative period yielded the early significant improvements in the Mayo Wrist score. The DASH score contains 38 questions, including different kinds of daily activities, highly strained and technically demanding works, which needs longer time of physiotherapy and mainly depend on patient’s subjective feedback. In our opinion, the DASH score is more suitable to evaluate functional recovery 12 months after scaphoid nonunion surgery.

This study has several limitations. First, our study had no control group; however, our results were comparable to Oh. et al., which had better carpal alignment but similar wrist function to the arthroscopic group, though the clinical results were similar [25]. Second, our study’s 12-month follow-up time was relatively short; late complications such as arthritis and screw migration may not be detected in this limited time. Finally, our study’s case number was relatively small. The findings in our study should be confirmed in a future study with a larger population.

## Conclusion

The surgical outcomes for scaphoid nonunion treated with combined volar bone grafting and dorsal antegrade headless screw achieved a high union rate and great wrist functional and radiographic outcomes. The earliest recovered wrist functional parameters were grip strength, Mayo Wrist score and motion arc at postoperative 6 months and finally the DASH score at postoperative 12 months. We believe that our findings are informative for clinical hand surgeons to predict the postoperative functional recovery course. Our findings also provide a reference for common functional scores at different evaluation times; however, these findings should be confirmed in a future study with a larger population and longer follow-up time.

## Data Availability

The authors agree for the publication and data usage.

## Abbreviations

A: Arthroscopy cancellous bone graft
O: Open volar cancellous bone grafting
SAL: Scaphoid axial length
LISA: Lateral intrascaphoid angle
SLA: Scapholunate angle
RLA: Radiolunate angle
CHR: Carpal height ratio
Pre: Preoperative degree
Post: Postoperative degree
NSD: No significant difference
ND: No data
K wire: Kirschner wire
AVN: Avascular necrosis
DISI: Dorsal intercalated segment instability
DASH: Disabilities of the Arm, Shoulder and Hand Score
VAS: Visual Analogue Scale
SD: Standard deviation
